# Lung Transplantation: CT Assessment of Chronic Lung Allograft Dysfunction (CLAD)

**DOI:** 10.3390/diagnostics11050817

**Published:** 2021-04-30

**Authors:** Anne-Laure Brun, Marie-Laure Chabi, Clément Picard, François Mellot, Philippe A. Grenier

**Affiliations:** 1 Radiology Department, Hôpital Foch, 92150 Suresnes, France; ml.chabi-charvillat@hopital-foch.com (M.-L.C.); f.mellot@hopital-foch.com (F.M.); 2 Respiratory Department, Hôpital Foch, 92150 Suresnes, France; c.picard@hopital-foch.com; 3 Department of Clinical Research and Innovation, Hôpital Foch, 92150 Suresnes, France

**Keywords:** lung transplantation, chronic lung allograft dysfunction, computed tomography (CT) scan

## Abstract

Chronic lung allograft rejection remains one of the major causes of morbi-mortality after lung transplantation. The term Chronic Lung Allograft Dysfunction (CLAD) has been proposed to describe the different processes that lead to a significant and persistent deterioration in lung function without identifiable causes. The two main phenotypes of CLAD are Bronchiolitis Obliterans Syndrome (BOS) and Restrictive Allograft Syndrome (RAS), each of them characterized by particular functional and imaging features. These entities can be associated (mixed phenotype) or switched from one to the other. If CLAD remains a clinical diagnosis based on spirometry, computed tomography (CT) scan plays an important role in the diagnosis and follow-up of CLAD patients, to exclude identifiable causes of functional decline when CLAD is first suspected, to detect early abnormalities that can precede the diagnosis of CLAD (particularly RAS), to differentiate between the obstructive and restrictive phenotypes, and to detect exacerbations and evolution from one phenotype to the other. Recognition of early signs of rejection is crucial for better understanding of physiopathologic pathways and optimal management of patients.

## 1. Introduction

More than 4500 lung transplantations (LTs) are performed every year worldwide. Despite global improvements in surgical techniques and post-operative management of patients, long-term outcomes after lung transplantation remain poor compared to those of other solid organ transplantation. According to the last International Society for Heart and Lung Transplantation (ISHLT) registry report, median survival in the most recent era of lung transplantation is 6.5 years [1]. The first cause of death remains graft failure/chronic rejection [1], clinically presenting as progressive pulmonary function decline. Graft failure affects up to 50% of the patients at 5 years post-transplantation and is responsible for reduced survival and reduced quality of life. 

For a long time, bronchiolitis obliterans syndrome (BOS) has been considered the hallmark of chronic allograft rejection. Different phenotypes have emerged in recent literature, and the division became more apparent when Sato et al. defined a restrictive phenotype affecting up to 30% of patients, with evidence of functional decline, named Restrictive Allograft Syndrome (RAS) [2]. The term Chronic Lung Allograft Dysfunction (CLAD) has been proposed to encompass the different phenotypes of chronic lung transplant rejection [3]. If the diagnosis of CLAD relies on spirometry, computed tomography (CT) has proven to be an important complementary tool in the diagnosis and characterization of the different subtypes [4]. The objectives of this review are to describe CT features associated with the different phenotypes of CLAD as they have been defined in recent international guidelines and to present potential future applications of CT that could improve the management of patients in this challenging area.

## 2. Definition of Chronic Lung Allograft Dysfunction (CLAD) 

CLAD is defined as a persistent and irreversible decline in forced expiratory volume in 1 s (FEV1) of at least 20% compared to the mean of the two best post-operative values at least 3 weeks apart.

**Figure 1:**
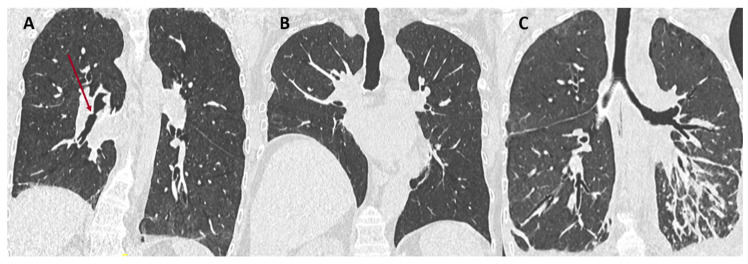
Examples of non-CLAD conditions (red arrow). (**A**) Right lower lobe bronchial stenosis responsible for obstructive changes on PFT; (**B**) post-surgical right phrenic nerve damage responsible for restrictive changes; (**C**) development of Kaposi’s sarcoma on the lung graft responsible for mixed obstructive and restrictive changes.

CLAD can present either as a predominantly obstructive pattern, a restrictive pattern, or a mixed obstructive and restrictive pattern that is not explained by other conditions. Numerous processes and diseases may be responsible for acute or chronic functional deterioration and are not included in the current definition of CLAD (Table 1). CT is very useful in identifying many of these causes (airway stenosis, phrenic nerve palsy, disease recurrence on the allograft, etc.) and thus plays an important role in the differential diagnosis [3,5]. Examples of conditions responsible for FEV1 decline, not related to CLAD, are illustrated in Figure 1. 

## 3. Diagnosis of CLAD

According to the last consensus report from the Pulmonary Council of the ISHLT, the critical level of ≥20% fall from baseline FEV1, with or without change in forced vital capacity (FVC), qualifies for “possible CLAD” and should prompt investigations to identify potentially treatable causes, such as infection, acute cellular/antibody-mediated rejection, or airway stenosis [5]. If lung function parameters remain impaired on a second reading at least 3 weeks after the first ≥20% fall from baseline and after adequate treatment has been given, a diagnosis of “probable” CLAD can be made [5]. CLAD severity (Table 2) and preferential phenotype (Table 3) should be defined at this stage to stratify potential investigations and therapies [6].

CLAD encompasses two main clinical entities, BOS and RAS, which can be differentiated on the basis of spirometry and imaging. RAS is characterized by persistent opacities on chest imaging (chest X-ray and/or CT) and a decline in total lung capacity (TLC) ≥ 10% compared to the best post-operative TLC [2] or a decline in FVC ≥ 20% compared to the best FVC when TLC is not available [6,7,8]. Lung volumetry obtained by CT can be used when plethysmography is not available [8,9]. BOS is characterized by a purely obstructive pattern (FEV1/FVC ratio < 0.7 and normal TLC) and no pulmonary opacity on chest imaging. Evidence of an obstructive and restrictive profile (FEV1/FVC < 0.7 and low TLC) associated with pulmonary opacities defines the mixed phenotype. Some patients remain undefined after functional and radiological evaluation [5,6,7,8,9,10]. The criteria for CLAD phenotypes are summarized in Table 3.

CLAD will be “confirmed” if the physiologic abnormalities persist for 3 months after the first value has been obtained. Further investigation, including CT, to exclude any treatable causes or complications of therapy may be warranted at any stage.

Azithromycin-Reversible Allograft Dysfunction (ARAD) is a form of functional decline that responds to neomacrolid azithromycin therapy [3,11,12,13]. As a reversible condition, it should not be considered part of CLAD. Also known as neutrophilic reversible allograft dysfunction, ARAD is characterized by the presence of neutrophils in bronchoalveolar lavage and a functional obstructive profile mimicking BOS [13]. However, the hallmark of ARAD is the resolution of the abnormalities with azithromycin [14,15]. Two randomized clinical trials have demonstrated that a prophylactic treatment with azithromycin was associated with a significant reduction of the prevalence of BOS [16,17]. 

## 4. Bronchiolitis Obliterans Syndrome

BOS is the most common form of CLAD, accounting for approximately 65–70% of cases and occurring in 48% of patients 5 years after lung transplantation [15]. It is characterized by irreversible small airway obstruction due to fibrosis and an obstructive physiologic profile. Risk factors for the development of BOS include acute rejection, primary graft dysfunction, lymphocytic bronchiolitis, infection/colonization with micro-organisms (*Pseudomonas aeruginosa*, *Aspergillus fumigatus*), donor and recipients genetic factors, and the presence of HLA antibodies or antibodies to self-antigens [10].

The pathophysiologic mechanism involves diverse immune pathways, which ultimately converge to the development of fibroproliferative tissue obstructing the small airways. At histopathology, there is partial or complete obstruction of bronchiolar lumens by a dense infiltrate made of smooth-muscle cells, myofibroblasts, and mature collagen [18]. Due to the heterogeneous distribution of BOS, transbronchial biopsies can be falsely negative, and tissue sampling is not mandatory for the diagnosis [19].

**Figure 2:**
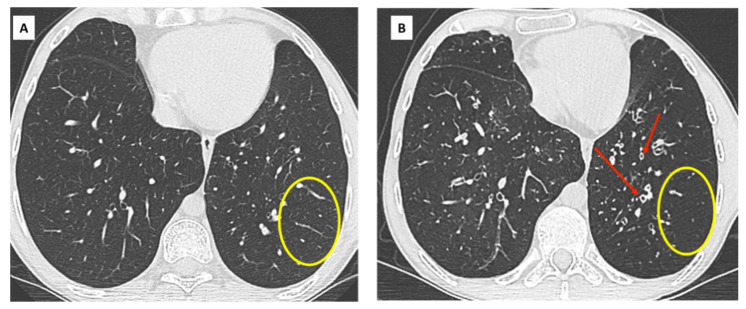
A 32-year-old man, bilateral LT in 2009 for cystic fibrosis. Diagnosis of BOS in 2011. (**A**) CT scan in January 2012 (FEV1 48%) shows no evidence of small or large airway disease; (**B**) CT scan in December 2017 (FEV1 18%, CLAD 4). Severe and diffuse bronchial wall thickening (red arrows), associated with mild bronchiectatic changes. Note the paucity of vascular markings in 2017 (yellow circles) compared to 2012 and subsequent hypoattenuation of lung parenchyma, related to small airway hypoventilation and fibrosis.

CT features of BOS include mosaicism of lung attenuation and expiratory air trapping, sometimes associated with large airway abnormalities. Mosaicism and air trapping are direct consequences of small airway fibrosis. Hypoventilation due to obliterans bronchiolitis is responsible for hypoxic vasoconstriction and manifests on a CT scan as low attenuation areas with small and rare vascular markings (Figure 2). 

**Figure 3:**
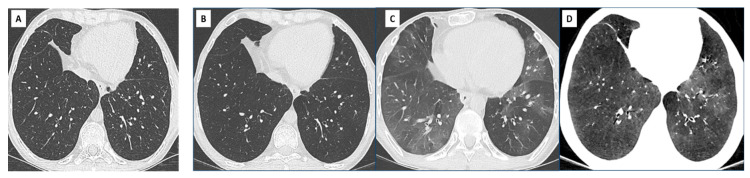
BOS in a 42-year-old patient. CT scans at CLAD diagnosis (**A**) and 2 years later (**B**–**D**); (**A**) August 2018: CLAD is diagnosed on spirometry with an obstructive profile. The CT scan does not show significant abnormality, confirming the BOS phenotype; (**B**) June 2020: inspiratory CT scan. Although the density of the lung parenchyma looks rather homogeneous, global hypoperfusion is obvious when comparing this CT to the baseline scan. The vessels are smaller and the overall density of the lung parenchyma is lower. There is no significant bronchial wall thickening nor large airway abnormality; (**C**) June 2020: expiratory scan unveils mosaicism and heterogeneous air trapping related to BOS; (**D**) June 2020: minimum intensity projection (mIP) technique applied on the inspiratory images (soft tissue kernel, parenchymal windowing) improves detection of mosaic perfusion with good correlation with expiratory images. This technique can be helpful in the absence of expiratory images or if the expiration has not been optimal.

These poorly perfused areas alternate with areas of relative hyperattenuation due to vascular redistribution in the spared lung [20]. In equivocal cases, expiratory CT may be used to differentiate air trapping from other causes of mosaic attenuation, such as occlusive vascular and infiltrative lung diseases [21,22,23]. In the absence of an expiratory CT scan, mosaicism can be depicted by minimum intensity projection (mIP) techniques applied on lung images reconstructed with soft tissue kernels (Figure 3). 

Large airway abnormalities, including bronchial wall thickening and mild cylindrical bronchiectasis, are often present [20]. Small centrilobular nodules and tree-in-bud may also be observed, related to bronchiolar impaction and/or wall thickening. It is important to exclude infection before attributing these abnormalities to BOS alone. The presence and severity of air trapping are closely related to functional obstruction parameters on spirometry. However, in the absence of functional abnormalities, CT has a poor sensitivity to diagnose BOS [24].

## 5. Restrictive Allograft Syndrome (RAS)

RAS is the second most common form of allograft rejection, with a prevalence of approximately 25–35% in the CLAD population. The outcome in patients with RAS is worse than patients with BOS, with a median survival of 6–18 months after the diagnosis of RAS compared to 3–5 years after the diagnosis of BOS [25,26].

RAS is defined by a restrictive pulmonary defect without evidence of obstruction and the presence of persistent opacities on chest imaging. Functional decline may be progressive and steady or marked by periodic stepwise exacerbations. Patients can also present with acute respiratory failure. The gradually progressive decline is associated with the best prognosis [5,27,28]. 

**Figure 4:**
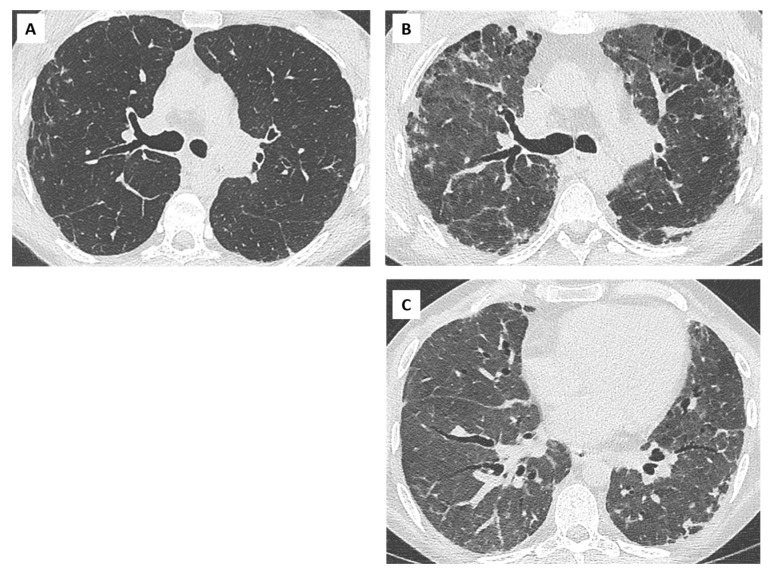
Diffuse opacities associated with RAS in a 38-year-old woman showing slowly progressive clinical symptoms and functional decline. (**A**) CT scan at CLAD onset, showing subtle reticular changes in the right upper lobe; (**B**) 2 years later, the patient has developed upper and subpleural predominant consolidation and reticulations, associated with paraseptal emphysema. Note the diffuse ground glass opacities consistent with inflammation and a possible NSIP pattern (**C**).

RAS has complex histopathologic features involving interstitial and small airway compartments. Fibrotic changes most often consist in intra-alveolar fibroelastosis (IAFE) adjacent to the bronchioles, the pleura, and the interlobular septa [29,30]. This pattern of IAFE is virtually identical to that observed in idiopathic pleuro-parenchymal fibroelastosis (PPFE), but patients with RAS often demonstrate associated lesions of obliterative bronchiolitis and the two diseases also display vascular differences [31]. In a study of RAS explant lungs using CT and microCT, investigators demonstrated large areas of disappearing airways, with obliterative bronchiolitis in 30–40% of the remaining airways. MicroCT showed a decrease in the number of terminal bronchioles [32]. Other fibrotic patterns encountered in RAS include non-specific interstitial pneumonia (NSIP) and fibrosis-induced paraseptal emphysema, the latter being associated with relatively good prognosis [30] (Figure 4). 

Fibrotic lesions can merge with areas of diffuse alveolar damage (DAD) and acute fibrinoid organizing pneumonia (AFOP). AFOP is characterized by intra-alveolar filling of ball-like fibrin exudates, without hyaline membranes, which would suggest a diagnosis of DAD, and without overt fibroblastic organization, which would suggest organizing pneumonia. It is considered a severe form of acute lung injury and is associated with a poor prognosis [33]. Whether and how these acute phenomena predispose to the development of fibrosis is still unclear, but some authors have suggested a temporal sequence between DAD [29] or AFOP [34] and IAFE. 

**Figure 5:**
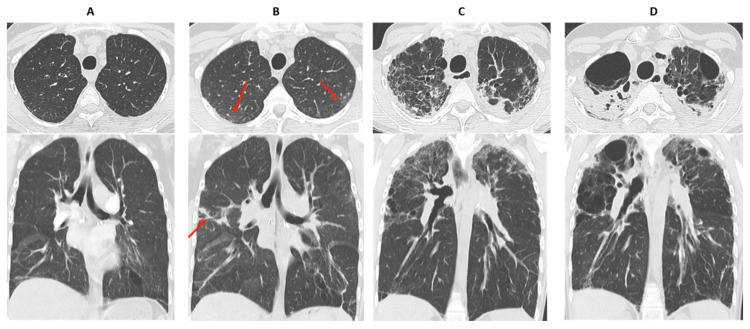
Early detection and evolution of RAS in a 24-year-old man, who underwent bilateral LT in 2007 for cystic fibrosis. Axial and coronal images of CT scans at optimal spirometry (**A**) 4 months before the diagnosis of CLAD (**B**), 8 (**C**) and 22 months (**D**) after the diagnosis of CLAD. (**A**) May 2008: normal CT scan; (**B**) August 2008: first visualization of subtle subpleural ground glass areas and consolidation (red arrows), considered nonspecific and treated with antibiotics. The diagnosis of CLAD was confirmed in December 2008 with a RAS profile; (**C**) August 2009: rapid evolution towards destructive, upper predominant cystic lesions, and interstitial fibrosis; (**D**) October 2010: late RAS. CT changes suggestive of pleuroparenchymal fibroelastosis with dense subpleural fibrosis and pleural thickening; dramatic loss of volume of the upper lobes.

Parenchymal abnormalities can precede the diagnosis of CLAD. Peripheral consolidation and peripheral ground glass have been reported in approximately 40% and 20% of cases, respectively, before functional confirmation of RAS [35]. Initial CT findings may be subtle and nonspecific (septal lines, ground glass nodules, focal consolidation, etc.), especially in immunosuppressed patients prone to infection, but their persistence is suspicious for RAS [27,36] (Figure 5).

Imaging features of late RAS often correlate with those of idiopathic PPFE [37]. They include pleural thickening, dense peripheral consolidations, traction bronchiectasis, architectural distortion, and volume loss, typically upper predominant and variable in extent. Some patients may present with diffuse or basal predominant interstitial abnormalities [9,38], and these patterns could be associated with a worse prognosis [38]. 

**Figure 6:**
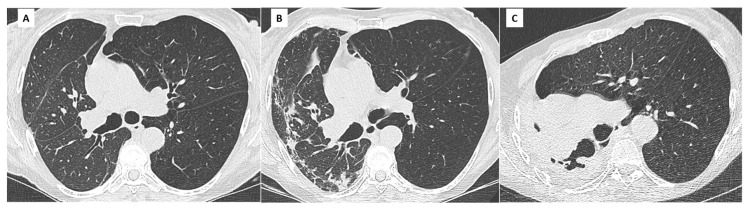
Unilateral RAS in a 61-year-old woman, who underwent right LT for α1-antitrypsin deficiency. (**A**): Baseline CT scan. Note the diffuse hypo attenuation and hypoperfusion of the left native lung, compared to the right allograft. An adapted normal FEV1 must be defined in this patient, taking into account the severe obstruction from the left lung; (**B**) Diagnosis of CLAD, with concomitant apparition of subpleural interstitial opacities in the right lung; (**C**) 3 years later, the allograft has been replaced by dense fibrosis and is totally collapsed. There is marked-over expansion of the left lung.

In a study comparing CT scans obtained at different time points (pre-CLAD, CLAD onset, post-CLAD, and late-CLAD), CT scores demonstrated a significant increase in bronchiectasis, central, and peripheral consolidation, architectural distortion, volume loss, and hilus retraction over time [35] (Figure 5 and Figure 6). The absolute FVC decrease post-CLAD diagnosis correlated with CT alterations. In another study, severe and multilobar consolidation on CT were associated with a reduced TLC and a poor prognosis [9]. 

**Figure 7:**
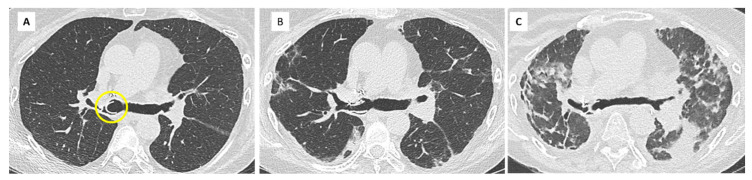
Acute exacerbation of RAS in a 67-year-old woman; (**A**) baseline CT scan. Stable obstructive pattern on PFT related to right main bronchus stenosis, treated by endo-bronchial stenting (yellow circle); (**B**) diagnosis of CLAD with restrictive profile. CT shows persistent subpleural opacities (ground glass mostly), consistent with RAS; (**C**) 1 month later, the patient presented with acute clinical and functional deterioration. CT shows extensive and rapidly progressive ground glass opacities, suggesting diffuse alveolar damage and/or AFOP. The patient died a few days later.

Extensive consolidation, ground glass opacities, and septal lines are suggestive of underlying DAD or AFOP lesions (Figure 7). Dubbeldam et al. described two distinct groups of patients according to the evolution of CT findings, a rapidly progressing group, possibly overlapping with AFOP lesions, and a more slowly progressing group, overlapping with PPFE and upper lobe fibrosis [27]. 

F-fluorodeoxyglucose positron emission tomography with computed tomography (F-FDG PET/CT) may help in differentiating BOS from RAS, as reported in a recent monocentric retrospective study [39]. Maximum standardized uptake value (SUV) was higher in RAS compared with BOS and stable patients (*p* < 0.0001), and high SUV in RAS patients was associated with worse survival. In the same study, the authors examined the explanted lungs using microCT and showed extensive fibrosis in regions of high SUV, with an increased number of glucose transporter-1 positive cells.

## 6. Overlap BOS/RAS: Mixed-Phenotype CLAD

**Figure 8:**
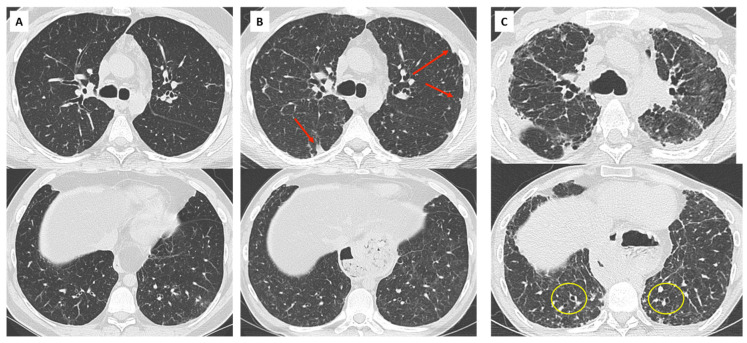
Mixed CLAD in a 30-year-old patient. (**A**) Diagnosis of CLAD in 2007 with initial obstructive phenotype. CT shows mild bronchial wall thickening; (**B**) progressive decline in TLC, suggesting an evolution towards a mixed CLAD (oct 2009). Note the apparition of subpleural opacities in the upper lobes (red arrows) and mild and diffuse ground glass opacities; (**C**) rapid functional and radiological deterioration, with upper predominant fibrosis and subpleural consolidation suggesting PPFE, mild cylindrical bronchiectasis in the lung bases (yellow circles).

The initial phenotype of CLAD should be re-assessed regularly as it can transition from one phenotype to another [2]. The combination of obstruction, restriction, and persistent lung opacities is usually defined as the mixed phenotype of CLAD [4]. Some patients may present with a mixed phenotype ab initio, whereas others may demonstrate a shift from the original phenotype (usually BOS) to a mixed phenotype over time [36] (Figure 8). In a recent study by Verleden et al., including 268 lung transplant patients with CLAD (18% RAS ab initio, 80% BOS, and 2% undefined), 25 patients (9%) developed a mixed CLAD phenotype (24 BOS to mixed-phenotype and 1 RAS to mixed-phenotype) [40]. This mixed phenotype was associated with apical predominance of CT opacities, PPFE on pathology, a lower FEV1 and FEV1/FVC at diagnosis compared to RAS ab initio, and similar survival compared to RAS ab initio patients. The BOS to mixed phenotype evolution was more frequently observed in patients transplanted for emphysema. 

## 7. When to Perform a CT Scan in the Setting of CLAD

An initial CT scan is recommended for all patients 6 months after lung transplantation, when spirometry is expected to be optimal [5]. It should be performed without iodine contrast, in high resolution (maximum width of 3-mm sections), at full inspiration and expiration. Repeat CT studies should be performed when a first drop in FEV1 ≥ 10% is seen to look for an identifiable cause (infection, drug toxicity, acute rejection, malignancy, etc.), and at CLAD onset to define the phenotype and as baseline imaging. 

Dettmer et al. demonstrated the utility of CT as a predictor of restrictive CLAD and predictor of survival [41]. CT at CLAD onset showed significantly more opacities in patients who later developed RAS, and a radiological score developed for inflammation showed significant correlation with survival. Lung volumetry obtained by CT can also help to differentiate BOS from RAS, as the volume of lungs in RAS is significantly lower compared to the baseline, while the volume of lungs in BOS remains stable or even increases [8]. 

During follow-up and according to clinical and functional changes, CT may be used to exclude any treatable causes or complications of therapy or to detect possible evolution to a mixed phenotype. 

## 8. Use of Artificial Intelligence (AI) in pre-CLAD and CLAD

Is machine learning (ML) able to detect CLAD earlier and with higher sensitivity than the current methodologies? 

Volumetric quantitative CT analysis has shown that, in patients with BOS, a decline in lung function was associated with a statistically significant increase in airway lumen area and a decrease in vessel cross-sectional area [42]. 

In a retrospective study including 178 LT patients, Barbosa et al. established quantitative CT (QCT) scores, including lung volumes and air trapping volumes by lobe, and semi-quantitative scores (absent, mild, moderate, severe) for features including mosaic attenuation and bronchiectasis on paired inspiratory and expiratory CT acquisitions [43]. QCT metrics demonstrated stronger correlations with FEV1 and, therefore, were better predictors of pulmonary function than semi quantitative scores. In unilateral LT, models using QCT metrics alone outperformed models using semi quantitative scores or PFT to diagnose BOS, and adding QCT metrics to PFTs improved diagnosis accuracy for all transplant types [44], suggesting that CT could allow early diagnosis of BOS, especially in single LT patients. The same authors assessed ML algorithms, utilizing QCT to predict eventual development of BOS [45]. Twenty-three baseline quantitative CT parameters, including lung volumes at lobe levels, central and distal airway volume, surface, and resistance, could significantly distinguish between non-BOS patients and eventual BOS developers (*p* < 0.5), whereas no spirometric parameter could. ML methods could identify BOS developers at baseline with an accuracy of 85%, using only three QCT parameters [45].

Using a biomechanical model-based platform to characterize changes in lung deformation, Horie et al. reported increasing lung deformation in patients with RAS compared to patients with BOS and no-CLAD patients [46]. They also measured quantitative density metrics (QDM) from CT density histograms (left and right quantile weights calculated from the left and right sides of the histogram) and showed that higher QDM values (reflecting higher density of the lungs) were associated with RAS and a lower survival [47]. The same authors reported later that higher QDM values at the time of early drop in FEV1 (10–19% drop from baseline) could predict CLAD in both single and double lung transplantation patients [48].

## 9. Conclusions

CLAD remains a major limiting factor for long term graft viability. CT in combination with spirometry plays a crucial role in the positive and differential diagnosis of CLAD. It helps identify the main phenotypes (BOS, RAS, and mixed) of CLAD, their evolution, and the intercurrent complications. Accurate diagnosis of the different phenotypes of CLAD is important to better understand the underlying pathogenesis, identify homogeneous populations for clinical trials, and guide future therapeutic approaches. Furthermore, quantitative imaging and IA-based algorithms are promising tools for early detection of CLAD and prediction of disease.

## Figures and Tables

**Table 1:** What is not included in the definition of CLAD (adapted from [5]).

(A) Processes and diseases responsible for chronic lung function decline. Stability for at least 6 months allows recalculation of a new baseline FEV1:
Decreasing lung function due to a normal ageing process; Parenchymal or chest wall surgery, phrenic nerve damage; Airway or vascular stenosis, chronic pleural effusion, persistent pulmonary edema; Post infective or postoperative chronic scarring; Donor/recipient mismatch; Weight gain; Disease recurrence on the allograft from the underlying transplant indication Drug toxicity.
(B) Processes and diseases responsible for a potentially reversible functional decline:
Any from (A) above where treatment achieves improvement of FEV1; Infection; Acute cellular/antibody-mediated rejection; Aspiration/gastro-oesophageal reflux; ARAD: Azithromycin-Reversible Allograft Dysfunction.

**Table 2:** CLAD staging (from [5]).

Stage	Spirometry
CLAD 0	FEV1 > 80% FEV1 baseline
CLAD 1	FEV1 > 65–80% FEV1 baseline
CLAD 2	FEV1 > 50–65% FEV1 baseline
CLAD 3	FEV1 > 35–50% FEV1 baseline
CLAD 4	FEV1 ≤ 35% FEV1 baseline

**Table 3:** CLAD phenotypes (from [5]).

KERRYPNX	Obstruction (FEV1/FVC < 0.7)	Restriction (TLC Decline ≥ 10% from Baseline)	Opacities on Chest X ray/CT
BOS	Yes	No	No
RAS	No	Yes	Yes
Mixed	Yes	Yes	Yes
Undefined	Yes	No	Yes
	Yes	Yes	No
